# Selenoprotein: Potential Player in Redox Regulation in *Chlamydomonas reinhardtii*

**DOI:** 10.3390/antiox11081630

**Published:** 2022-08-22

**Authors:** Sandip A. Ghuge, Ulhas Sopanrao Kadam, Jong Chan Hong

**Affiliations:** 1 Agricultural Research Organization (ARO), The Volcani Institute, P.O. Box 15159, Rishon LeZion 7505101, Israel; 2 Division of Life Science and Division of Applied Life Science (BK21 Four), Plant Molecular Biology and Biotechnology Research Center, Gyeongsang National University, Jinju 52828, Gyeongnam, Korea; 3 Division of Plant Sciences, University of Missouri, Columbia, MO 65211, USA

**Keywords:** selenoprotein, selenocysteine, antioxidants, algae, Chlamydomonas

## Abstract

Selenium (Se) is an essential micro-element for many organisms, including *Chlamydomonas reinhardtii*, and is required in trace amounts. It is obtained from the 21st amino acid selenocysteine (Sec, U), genetically encoded by the UGA codon. Proteins containing Sec are known as selenoproteins. In eukaryotes, selenoproteins are present in animals and algae, whereas fungi and higher plants lack them. The human genome contains 25 selenoproteins, most of which are involved in antioxidant defense activity, redox regulation, and redox signaling. In algae, 42 selenoprotein families were identified using various bioinformatics approaches, out of which *C. reinhardtii* is known to have 10 selenoprotein genes. However, the role of selenoproteins in Chlamydomonas is yet to be reported. Chlamydomonas selenoproteins contain conserved domains such as CVNVGC and GCUG, in the case of thioredoxin reductase, and CXXU in other selenoproteins. Interestingly, Sec amino acid residue is present in a catalytically active domain in Chlamydomonas selenoproteins, similar to human selenoproteins. Based on catalytical active sites and conserved domains present in Chlamydomonas selenoproteins, we suggest that Chlamydomonas selenoproteins could have a role in redox regulation and defense by acting as antioxidants in various physiological conditions.

## 1. Introduction

Many organisms, including bacteria and humans, require Selenium (Se) as an essential trace element. Se is known to have various roles essentially related to redox homeostasis. Moreover, Se in trace amounts provides various health benefits such as improving immunity and preventing heart diseases and cancer [1,2,3,4,5]. Selenocysteine (Sec, U), the 21st amino acid, is Se’s main biologically active and available form. Sec is encoded by the UGA stop codon followed by co-translational insertion into selenoproteins in response to various elements involved in the Sec biosynthesis pathway. Selenoproteins are present in animals, bacteria, and archaea, whereas fungi, higher plants, and yeast lack selenoproteins. However, plants have cysteine-containing homologs of selenoprotein (hereafter referred to as Cys-homologs) [6]. 

Sec biosynthesis is highly complex, follows a unique translation process compared to other amino acid syntheses, and requires various *cis*- and *trans*-acting elements [7,8,9,10,11]. The Sec biosynthesis pathway is different in prokaryotes and eukaryotes. Here, we focus on Chlamydomonas selenoproteins; hence, we have covered the Sec biosynthesis pathway of eukaryotes only in the next part. In all organisms containing selenoproteins, the Sec biosynthesis starts with serylation of a specialized tRNA^Sec^, catalyzed by a seryl-tRNA synthetase (SerRS). Next, *O*-phosphoseryl-tRNA^Sec^ kinase (PSTK), phosphorylates the seryl moiety of Sec (Ser-tRNA^Sec^) followed by replacement of the phosphoryl group with a selenol moiety by *O*-phosphoseryl-tRNA:selenocsteinyl-tRNA synthase (SepSecS) [12,13,14,15,16]. Here, selenol moiety is provided by the enzyme Sec lyase, which extracts Se from existing Sec, and is further used by selenophosphate synthetase (SPS2) to form selenophosphate [14,17]. Once Sec-tRNA^Sec^ is synthesized, translation of UGA requires Sec-specific elongation factor (eEFSec) and SECIS binding protein 2 (SBP2) to recruit Sec-tRNA^Sec^ to the ribosome in addition to the SECIS element [18,19,20]. SECIS is in the *cis*-element, forming a hairpin structure, and is present in selenoprotein in 3′-UTR [21,22,23,24]. In addition, after tRNA release, the ribosomal protein eL30 binds to SECIS and displaces SBP2 in eukaryotes and archaea [25]. 

## 2. Selenoproteins and Cysteine-Containing Homologs

**Figure 1:**
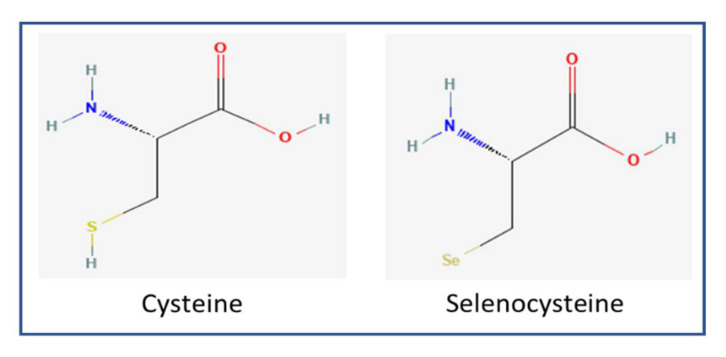
Chemical structure of cysteine (MW: 103.01 Da; PubChem CID: 5862) and selenocysteine (MW: 168.05 Da; PubChem CID: 6326983) [35].

Cysteine (Cys) is a principal sulfur-containing amino acid, while Sec is a Se-containing amino acid. Sec is structurally similar to Cys, except for the selenol group instead of the thiol group (Figure 1). Se and sulfur are chalcogens; thus, Sec and Cys share specific chemical properties. Nevertheless, there are slight differences in terms of electronegativity, oxidation state, and atomic radius between Se and sulfur. Se has a longer atomic radius and longer bond lengths than sulfur. Se clearly has unique chemical properties that differ from sulfur, but perhaps the similarities between the two elements are more striking. Slight differences in the electronic structures of sulfur and Se are enough to give selenoproteins distinctive catalytic potential [26,27]. Moreover, Sec has a lower pKa (~5.2) than Cys (~8.0); this way, it can exist as a nucleophile without electrostatic interactions, and hence, Sec is more reactive than Cys under physiological conditions [28]. Selenoproteins are more highly resistant to irreversible oxidation under severe oxidative stress conditions than their Cys-homologues [6,29]. Previous reports of Sec residue elimination, Sec residue alkylation at pH 6.5, and Sec substitution with Cys result in reduced catalytic efficiency of selenoprotein [30,31,32,33,34].

Human selenoproteins broadly can be classified into two groups based on the position of Sec amino acid in selenoprotein. In a group, I Sec is positioned at the C-terminal region, while in a group, II Sec is positioned at the N-terminal region. Group I includes thioredoxin reductases (TrxR), Selenoprotein I (SelI), SelK, SelO, SelR, and SelS, while Group II includes glutathione peroxidases (GPX), SelM, SelN, SelT, SelV, and SelW [36]. The human genome codes for 25 selenoproteins, most of which are involved in oxidative stress responses and redox signaling [2,37]. A common feature observed in Cys-homologues where Cys is present instead of Sec includes a conserved CXXC motif. CXXC motif alterations have been observed to affect the protein’s redox potential, its ability to function as a disulfide isomerase, and its interaction with folding protein substrates and oxidants [34]. Amongst human selenoproteins, SelV, SelW, Sep15, SelM, SelT, SelP, and others contain a CXXU motif instead of a CXXC motif at the Trx active site, indicating its antioxidant potential. Human selenoproteins contain Sec in the catalytic site identified for carrying out various redox functions, including redox signaling, antioxidant defense, and the regulation of redox homeostasis. However, there are also various selenoproteins from a human whose role is yet to know, including SelH, SelI, SelM, SelO, SelV, and SelW [36,37,38,39]. Isolation and quantification of selenoproteins are essential aspects due to its antioxidant properties. Isolation of selenoproteins, particularly selenoprotein P, is carried out using immunoaffinity precipitation, chromatography (using antibodies), chemical affinity, and immobilized metal affinity methods, which are further characterized using MS-based methods [40]. Recombinant selenoprotein production in *E. coli* is another widely used area for selenoprotein production. Mammalian TrxR selenoprotein obtains higher expression when expressed recombinantly in *E. Coli* with certain modifications [41,42]. For recombinant TrxR purification, a redox-active Sel-tag was developed. This tag can be used for one-step purification of tagged protein, selenolate-targeted fluorescent labeling, as well as selenolate-targeted radiolabeling can be purified using protocol [43,44].

## 3. Selenoproteins from Algae

Till 2002, selenoproteins were known only in animals. Interestingly ten selenoproteins were identified in *C. reinhardtii,* including methionine-S-sulfoxide reductase (MsrA), a selenoprotein specific to *Chlamydomonas* and not found in other organisms. Out of 10 Chlamydomonas selenoproteins, two selenoproteins, namely, GPX and SelW1, were identified at the protein level using mass spectrometry, while the rest of the amino acids were identified using genomic and EST databases. Moreover, the same study also reports that a selenocysteyl-tRNA (Sec tRNA) explicitly recognizes the UGA codon [45,46]. Further, more than 1000 selenoprotein genes from 42 selenoprotein families were predicted from genomic (36 organisms) and/or transcriptomic (including EST) datasets of 137 species of algae by using various bioinformatics approaches. Of them, 19 selenoproteins including AhpC, DsbA, SPS, GPX, DIO, TrxR, Sel F, Sel K, Sel M, MsrB, Sel S, Sel T, and SelW were also reported in animals [47,48]. GPX, TrxR, Sel U, and Sel T were the most abundant selenoproteins in algae, as these were found in more than half of the 36 genomes [47], while in specifically Chlamydomonas genome, GPX and SelW1 were the most abundant selenoproteins [45]. Further, we will discuss the known role of selenoproteins in humans, followed by the information available on selenoproteins from Chlamydomonas. We focused on Chlamydomonas selenoproteins only as these are identified by various groups [45,46,47].

### 3.1. Glutathione Peroxidase (GPX)

Selenoprotein glutathione peroxidase (GPX) is a cellular antioxidant enzyme that catalyzes the reduction of hydrogen peroxide, lipid hydroperoxides, and other organic hydroperoxides by oxidizing glutathione and, thus, helps to protect cells against oxidative damage [49,50]. The GPX family is widespread in living organisms, from archaea to bacteria to eukarya domains. In the case of mammals, five out of eight are selenoprotein GPX [51]. Loss of GPX activity is associated with muscle disorders [52], cancer [53,54], hepatopathies [55], renal failure [56,57], and neurological disorders such as Parkinson’s disease and Alzheimer’s disease [58,59,60]. It has been observed that mammalian selenoprotein GPXs have much higher activity than plant GPXs containing Cys [61]. Interestingly, mammalian selenoprotein GPX activity declines drastically when Sec is replaced by Cys [62]. The peculiar role of various selenoprotein GPXs are listed below in Table 1. In the case of Chlamydomonas, two selenoprotein GPX (GPX1-accession no. AY051144; GPX2- Gene Identifier from Phytozome- Cre08.g358525) were identified both containing Sec residue. Out of the two GPX selenoenzyme, in one GPX, the presence of Sec residue is confirmed at the proteomics level using mass spectrometry [45,46]. Moreover, its subcellular location is suggested in the mitochondria [46]. Another GPX is identified using Chlamydomonas EST and genomic databases [45].

### 3.2. Thioredoxin Reductase (TrxR)

**Figure 2:**
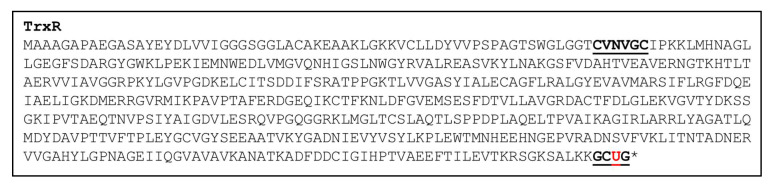
TrxR selenoprotein sequence from Chlamydomonas with conserved catalytic domains. Catalytic domain (CVNVGC and GCUG), underlined in bold; Sec, underlined bold red U; *—stop codon.

TrxRs are prominent selenoproteins enzymes that are known to regulate redox metabolism. Various studies suggest that human TrxR inhibits multiple stages of tumor progression [74,75,76]. Moreover, its loss or overexpression in humans is linked with the onset of several diseases, such as cancer, cardiovascular diseases, type II diabetes, neurological disorders, and human immunodeficiency virus infection [77,78,79,80]. Some of the important roles of TrxR are mentioned below in Table 1. Generally, TrxR contains an N-terminal redox center with a ‘CVNVGC’ conserved sequence and a C-terminal redox center with GCUG conserved amino acid sequence [81,82,83]. One TrxR selenoprotein (accession no. AF494052) was found in the Chlamydomonas genome [45,47]. Interestingly, TrxR from Chlamydomonas contains both conserved sequences at the N- and C-terminals with Sec amino acid residue C-terminal catalytic region (Figure 2).

### 3.3. Other Selenoproteins

**Figure 3:**
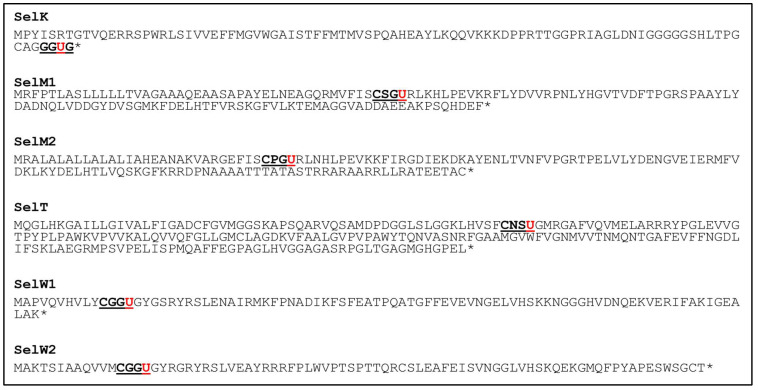
Selenoproteins sequence from Chlamydomonas with conserved catalytic domains. Catalytic domain (CXXU), underscore and bold; Sec (U), underscore and bold in red; *—stop codon.

In addition, to two GPXs and a TrxR, there are seven selenoproteins found in Chlamydomonas, namely, Selenoprotein (Sel) K (accession no. AAN32902), Sel M1 (accession no. AAN32905), Sel M2 (accession no. AAN32900), Sel T (Gene Identifier from Phytozome- Cre14.g616950, Sel W1 (accession no. AAN32901), Sel W2 (accession no. XP_001693902), and Chlamydomonas specific selenoprotein MsrA (accession no. AAN32904) [45]. Human SelT knockdown mutant induces expression of another selenoprotein gene Sel W along with oxidoreductase genes, indicating a role of SelT in redox regulation [84]. Moreover, in humans, SelT was found to prevent severe movement impairment in Parkinson’s disease by protecting dopaminergic neurons against oxidative stress [85]. However, most selenoproteins such as SelM and SelW have not been explored to find out its function. Interestingly Chlamydomonas, selenoproteins Sel M1, Sel M2, SelT, SelW1, and SelW2 contain a CXXU motif instead of the CXXC motif of the Trx active site (Figure 3), as they have been found in human selenoprotein, indicating its antioxidant potential [34].

## 4. Selenoproteins from Chlamydomonas Can Be Potential Antioxidants

It has been observed that Se is required for optimal growth of Chlamydomonas [45]. To the best of our knowledge, no reported study points out the role of selenoproteins in the Chlamydomonas and other algae until now. It has been observed that Sec biosynthesis machinery and selenoproteins containing Sec are present in Chlamydomonas but during evolution from the other amino acid biosynthesis pathways. Chlamydomonas and other green algae maintain all these enzymes in their genome, suggesting that selenoproteins might have some key roles. Selenoproteins found in Chlamydomonas share various similarities with selenoproteins from humans, including conserved regions, catalytic sites, and the presence of Sec instead of Cys in catalytic sites. In Chlamydomonas, most of the selenoproteins contain catalytically important CXXU region corresponding to the CXXC motif in the Trx active site. In contrast, TrxR contains both catalytically necessary conserved sequences at N-terminal and C-terminal redox center. It has been noted that alteration in the amino acid residues between two Cys affects redox potentials which shows the critical role of this catalytic site in redox regulation [86]. Chlamydomonas selenoproteins contain active catalytic sites required for redox functioning, indicating a potential role of selenoproteins as an antioxidant and in redox signaling in various physiological conditions, as was the case with human and other organism selenoproteins [36,37,38,39]. It has been observed that *Emiliania huxleyi* and *Aureococcus anophagefferens* algae have the most selenoprotein genes. Interestingly, both *algae* showed strong resilience against several environmental conditions such as wide temperature range, low light, and broad geographical distribution [87], which could be attributed to its extensive repertoire of selenoproteins. This study also indirectly supports the hypothesis that selenoproteins may be involved in various redox functions related to plant defense against stress growth conditions.

## 5. Conclusions and Future Perspectives

This opinion article is focused on various selenoproteins from *C. reinhardtii*. After the discovery of 10 selenoproteins in Chlamydomonas and knowing that Se is necessary for optimal growth of Chlamydomonas, no study has been reported suggesting the precise role of selenoprotein in Chlamydomonas. This area has a huge potential to find its roles in various physiological conditions. Such studies not only unravel the role of selenoproteins but will also provide information about the evolution process of selenoprotein, which would be very helpful in finding how and why selenoprotein is lost in higher plants. Moreover, if the Chlamydomonas selenoproteins are involved in oxidoreductase functions, it opens a wide area to make a climate-resilient plant system to combat various biotic and abiotic stress conditions using selenoproteins.

## Figures and Tables

**Table 1:** Functions of some of the selenoproteins.

Selenoprotein (Containing Sec Residue)	Functions	References
Glutathione peroxidase 1 (GPX 1)	As an antioxidant, also functions as Se storage house	[63,64]
GPX 2	As an antioxidant, anti-apoptotic function in the colon regulates mucosal homeostasis	[65]
GPX 3	As an antioxidant, preventing plasma LDL oxidation, functions in the reduction of H_2_O_2_	[66,67,68]
GPX 4	As an antioxidant protects brain membranes from peroxidative degradation, catalyzes the reduction of hydroperoxides, inhibits lipid peroxidation	[69,70,71,72]
GPX 6	Unknown	-
Thioredoxin reductases 1 (TrxR1)	As an antioxidant, reduction of thioredoxin, actin polymerization for cell membrane restructuring	[37,73]
TrxR2	Regulation of mitochondrial redox homeostasis, Maintains thioredoxin in a reduced state	[37]
TrxR3	Unknown	-

## Data Availability

This article presents the data generated and analyzed in figures or tables. Additional information on methods or materials used in this study will be made available upon request to the corresponding author.
